# A Review on Clostridioides Difficile Testing and How to Approach Patients With Multiple Negative Tests: A Case Report

**DOI:** 10.7759/cureus.34285

**Published:** 2023-01-27

**Authors:** Raghav Bassi, Pranav Prakash, Anuoluwa Oyetoran, Rabab Elsadek, Isaac Loseke, John R Leibach

**Affiliations:** 1 Internal Medicine, University of Central Florida College of Medicine, Graduate Medical Education/Hospital Corporation of America (HCA) Florida North Florida Hospital, Gainesville, USA

**Keywords:** multiple negative tests, c.diff testing, false-negative, pseudomembrane, c.diff colitis

## Abstract

*Clostridioides difficile* (*C. difficile*) is an important nosocomial infection that is commonly associated with antibiotic use with pseudomembranous colitis being present in only 13% of cases. Disease severity ranges from asymptomatic carriers to severe complicated disease, based on clinical and laboratory findings. There is no single rapid FDA-approved test to diagnose *C. difficile *infections (CDI) and diagnosis usually requires a multi-step diagnostic approach. *C. difficile *testing usually begins with the *C. difficile* toxin and glutamate dehydrogenase antigen screen (GDH). If testing is negative for either, then nucleic acid amplification testing (NAAT) is done to confirm the diagnosis. Endoscopic evaluation may be required in rare instances when there is a high clinical suspicion of disease with negative testing. Here, we present an interesting case of a patient with multiple negative *C. difficile *toxin and GDH tests. Given the high index of clinical suspicion of CDI, the patient underwent a colonoscopy which revealed diffuse pseudomembranous colitis. The patient was then appropriately treated with oral vancomycin. We aim to shed light on the different testing modalities available to clinicians and the indications for doing a colonoscopy to delineate between false positive testing and active CDI.

## Introduction

*Clostridioides difficile* (*C. difficile*) is an anaerobic toxigenic bacterium that is a leading cause of diarrhea in North America and Europe. According to a study published by the CDC in 2015, it was estimated that approximately half a million people are infected with *C. difficile *every year and it has a 5.8% mortality rate in 30 days [1]. The mortality is higher in older individuals with 1 in 11 patients above the age of 65 dying within 30 days of diagnosis [1].

Over the last two decades, there has been a significant increase in the incidence and severity of infections [1]. *C. difficile* is most commonly acquired in healthcare settings such as hospitals, outpatient clinics, and nursing homes. The use of antibiotics is responsible for a majority of *C. difficile *infections (CDI) with the most commonly implicated antibiotics being: penicillins, cephalosporins, and clindamycin [2]. Other etiologies include the use of proton pump inhibitors, previous *C. difficile* infection, chronic liver disease, chronic kidney disease, and malnutrition [2,3].

CDI associated with antibiotic use commonly presents as acute watery diarrhea in about 21% of patients [1,2]. Disease progression can lead to fulminant colitis, sepsis, and toxic megacolon with the formation of pseudomembranes lining the intestine in a mere 10% of cases [4]. In rare instances, complications such as protein-losing enteropathy and reactive arthritis have been reported in the literature [4]. Numerous testing modalities have been developed to diagnose CDI, with each test targeting a different component of the bacteria. These tests also have differing sensitivities, negative predictive values, and turnaround time, ultimately affecting how they are used in clinical practice. These tests include cytotoxicity assay which looks for free toxins in the stool and enzyme-linked immunoassay (EIA) which detects the presence of the bacteria by detecting levels of glutamate dehydrogenase (GDH) antigen in the stool. GDH is produced by *C. difficile* and is used to convert L-glutamate into α-ketoglutarate through an irreversible reaction, making it a highly effective screening tool [5]. Other tests available include toxigenic culture and nucleic acid amplification testing (NAAT), which are specific for the toxigenic *C. difficile* strains [5]. Effective detection of toxigenic *C. difficile* strains requires the use of a multimodal approach to rapidly diagnose symptomatic CDI while minimizing the probability of overdiagnosis of asymptomatic carrier states.

We present a rare case of a patient with evidence of active *C. difficile* colitis on colonoscopy despite multiple negative *C. difficile* tests. To our knowledge, this is one of the few cases in the literature of a patient who presented with severe complicated CDI despite having multiple negative stool antigen and toxin tests. Through this paper, we hope to go over the different screening tests available to clinicians and when to initiate empiric treatment despite negative *C. difficile* testing.

## Case presentation

A 79-year-old Caucasian male with a past medical history significant for pulmonary hypertension secondary to severe chronic obstructive pulmonary disease presented to the emergency department with a one-week history of worsening watery diarrhea, abdominal pain, and vomiting. The patient had seen his primary care doctor earlier in the week with the same symptoms and was prescribed metronidazole for suspected bacterial gastroenteritis, with minimal symptomatic improvement. The patient denied hematochezia, melena, unintentional weight loss, a history of travel outside the United States, and any other additional antibiotic use prior to initial symptom onset. The patient's last colonoscopy was more than 10 years ago which the patient reported as normal.

**Figure 1:**
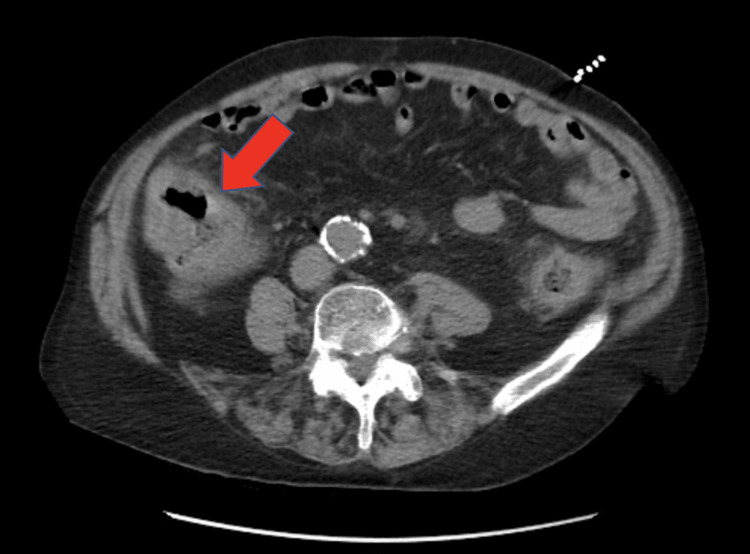
Computed tomography (CT) of the abdomen/pelvis without contrast on admission showing generalized large bowel inflammation with wall thickening and infiltration of the adjacent fatty tissue consistent with generalized colitis or inflammatory bowel disease

On presentation, the patient met sepsis criteria with a white cell (WBC) count of 20.3x10^9^/L (normal range: 4,500-11,000x10^9^/L). The chest X-ray showed bilateral lung infiltrates suspicious for pneumonia. Computed tomography (CT) of the abdomen/pelvis without contrast showed generalized large bowel inflammation with wall thickening and infiltration of the adjacent fatty tissue consistent with generalized colitis or inflammatory bowel disease (Figure 1). Blood cultures, stool cultures, and studies for *Giardia*, *Cryptosporidium*, *Shigella*, *Salmonella*, *Escherichia coli O157:H7*, and *Yersinia enterocolitica* were ordered given his history of recurrent episodes of watery diarrhea. The patient was started on intravenous metronidazole and levofloxacin for empiric coverage and admitted for further evaluation.

On hospital Day 2, the WBC count was trending up and was found to be 46.5x10^9^/L. The antibiotic regimen was broadened to piperacillin/tazobactam and intravenous vancomycin due to worsening leukocytosis. The patient began experiencing acute hypoxic respiratory failure and went into septic shock requiring vasopressor support. The patient was then transferred to the intensive care unit where a rectal tube was placed given his persistent diarrhea. Final blood cultures and stool cultures did not reveal any microbial growth. Stool studies were all unremarkable as well.

On hospital Day 7, the patient was hemodynamically stable but was still having continued loose stools in the rectal tube without any symptomatic relief. The infectious diseases team was consulted and they recommended holding antibiotics and obtaining a *C. difficile* EIA GDH antigen test and toxin A/B tests with reflex stool cultures. The *C. difficile* GDH antigen test and toxin A/B tests were both negative. The patient was started on intravenous metronidazole and repeat C. difficile testing with EIA GDH antigen and toxin assays were ordered due to a high index of clinical suspicion. However, these tests came back negative as well. Stool cultures did not reveal any growth so a colonoscopy was recommended for further evaluation. The general surgery team was consulted and they recommended a third set of *C. difficile* GDH antigen and toxin A/B testing for further confirmation before performing a flexible sigmoidoscopy.

**Figure 2:**
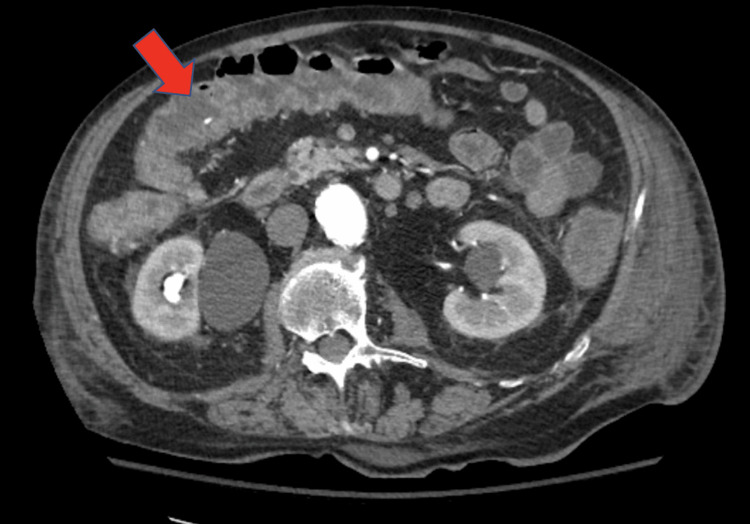
A repeat CT abdomen and pelvis on hospital day 9, which revealed worsening inflammatory changes in the sigmoid, descending, and ascending colon suggestive of colitis.

The patient’s third set of *C. difficile* screening was also negative; however, flexible sigmoidoscopy was withheld because the patient was deemed a high risk for sedation. Intravenous antibiotics were continued and the patient was transferred to our tertiary care center for higher acuity of care and where he could receive an evaluation from gastroenterology. Upon arrival at our facility, the patient was hemodynamically stable with a resolution of his pneumonia after receiving intravenous antibiotics. However, he was still having persistent watery diarrhea. A repeat CT abdomen and pelvis showed worsening inflammatory changes in the sigmoid, descending, and ascending colon suggestive of colitis (Figure 2). The gastroenterology team recommended stopping all previous antibiotics, and based on the high clinical suspicion for *C. difficile,* decided to treat with oral vancomycin until a repeat *C. difficile *toxin screening could be completed at this facility.

**Figure 3:**
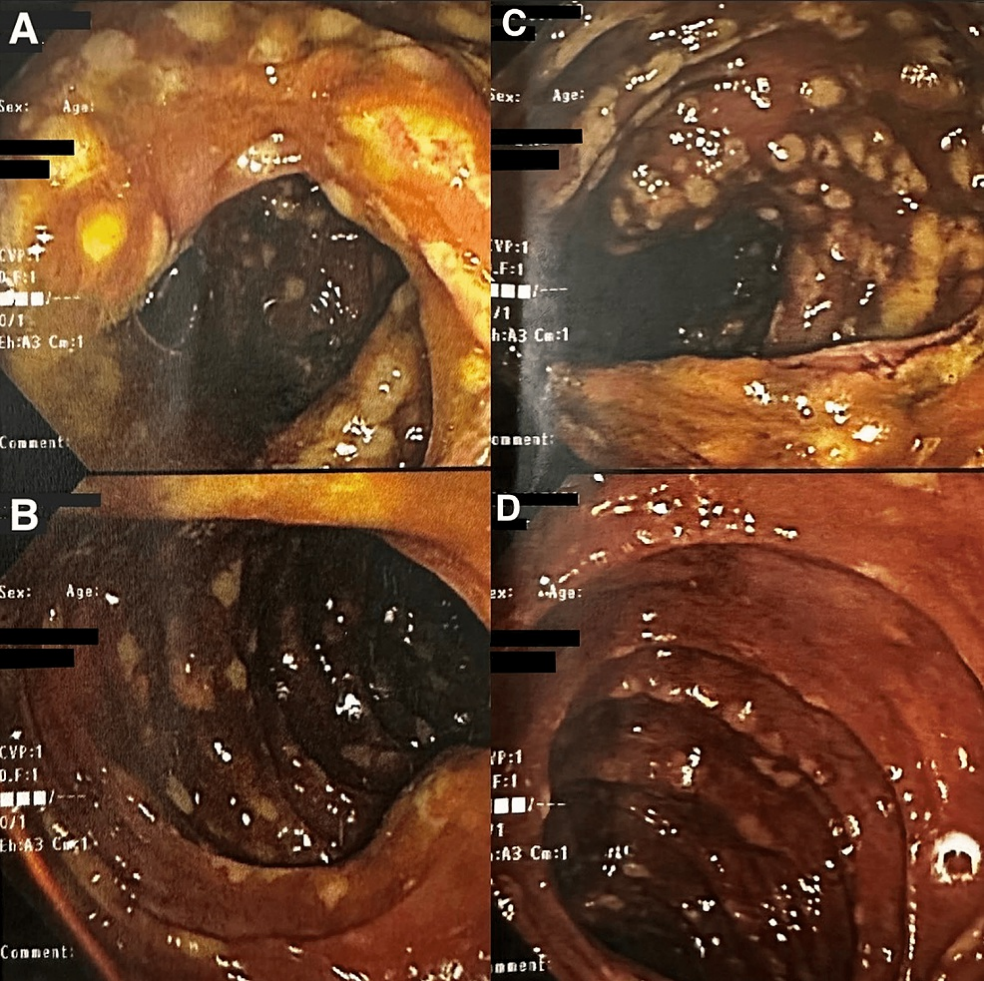
Colonoscopy revealing diffuse pseudomembrane formation throughout the colon. Images A and B reveal pseudomembrane formation in the transverse colon. Images C and D reveal pseudomembrane formation in the ascending colon.

The *C. difficile* toxin and GDH antigen screen were negative for the fourth time, so oral vancomycin was held due to increasing suspicion of antibiotic-associated diarrhea. The patient then underwent a colonoscopy for further evaluation which revealed diffuse pseudomembranous colitis throughout the entire colon (Figure 3). Biopsies were taken during endoscopic evaluation which confirmed CDI. The patient was restarted on oral vancomycin for a total of 14 days. Subsequently, the patient began to experience symptomatic improvement with a resolution of his diarrhea. His WBC count continued to trend down to 11.5x10^9^/L after oral antibiotic initiation. The remainder of the hospital stay was unremarkable and he was discharged home in improving and stable condition.

## Discussion

CDI is one of the most commonly recognized healthcare-associated infections in the United States. It has also been identified as a cause of community-associated diarrhea and traveler's diarrhea in vulnerable patient populations [6]. The disease spectrum ranges from non-severe to fulminant infection and death [7] where non-severe disease is characterized by watery diarrhea with three or more loose stools per day. It can also be associated with nausea and low-grade fevers in about 15% of cases [7]. As summarized in Table 1, three different severity criteria for CDI are outlined below based on clinical symptoms and laboratory values. According to the SHEA/IDSA and ACG guidelines, our patient met the criteria for severe complicated CDI. 

**Table 1:** Three different severity criteria for CDI based on the IDSA, hospital-specific guidelines and ACG guidelines ICU: intensive care unit; IDSA: Infectious Diseases Society of America; SHEA: Society for Healthcare Epidemiology of America, ACG: American College of Gastroenterology

Three different severity criteria for Clostridium difficile infection (CDI)			
Severity criteria	Mild-moderate disease	Severe disease	Severe complicated
Hospital-specific guidelines [15]	≥3 diarrheal stools/day; may be accompanied by mild or moderate abdominal discomfort, elevated WBC count, and fever	Mild-moderate criteria plus at least 1 of the following: (1) At least 3 of the following: temperature >38.3°C, WBC count >20,000 cells/mm3, albumin level <2.5 g/dL, age ≥65 years, ICU admission OR (2) Endoscopically or histologically confirmed pseudomembranous colitis OR (3) Toxic megacolon, perforation, colectomy, or septic shock requiring ICU admission and pressors	
SHEA/IDSA guidelines [16]	WBC count <15,000 cells/mm3 AND serum creatinine <1.5 × baseline	WBC count ≥15,000 cells/mm3 OR serum creatinine ≥1.5 × baseline.	Associated with hypotension or shock, ileus, or megacolon
ACG criteria [17]	Diarrhea with any additional signs or symptoms not meeting severe or complicated criteria	Albumin <3g/dL with either WBC ≥15,000 cells/mm3 or abdominal tenderness	Associated with admission to the ICU, hypotension, T≥ 38.5 °C, ileus or significant abdominal distention, mental status changes, WBC ≥35,000 cells/mm3 or <2,000 cells/mm, serum lactate >2.2 mmol/L or end organ failure

There are multiple mechanisms in the lifecycle of the bacterium that contribute to its virulence. The ability of the bacterium to form spores when exposed to the anoxic environment of the intestine plays a key role in its transmissibility, as the bacterium is able to survive in unfavorable conditions [8]. When these spores are exposed to bile acids in the intestine, they germinate and render the bacterium capable of invading the intestinal epithelium. The bacterium primarily uses two toxins to mediate its pathologic effects: toxin A and toxin B [8]. These toxins enable the bacterium to bind to cellular receptors and subsequently undergo receptor-mediated endocytosis. These toxins cause disruption of the cellular architecture and intercellular tight junctions, leading to the destruction of the integrity of the intestinal epithelium resulting in colitis [8].

There is no single rapid FDA-approved test to diagnose CDI and diagnosis usually requires a multi-step diagnostic approach [9]. The current management for the diagnosis and treatment of CDI starts with clinical suspicion. Patients typically present with acute onset diarrhea with ≥ 3 stools in a 24-hour period in the setting of recent antibiotic use and/or hospitalization, fever, abdominal pain, and leukocytosis [8,9]. Stool samples should be collected and sent for stool GDH antigen testing and EIA toxin A and B testing. If testing is negative for either, then a NAAT is done to confirm the diagnosis [10]. The EIA toxin A and B and GDH antigen combination testing are favored clinically because of their affordability, efficiency, and practicality [11]. A systematic review by Kazanowski et al. revealed that EIA detecting toxins A and/or B was about 95% specific making it a good screening test due to the low number of false positive results [12]. However, the test’s sensitivity was only about 70-80%, making them less effective in ruling out CDI. NAATs, on the other hand, have excellent sensitivities and specificities, but can result in overdiagnosis and overtreatment as they can detect CDI with no toxin production. Summarized in Table 2 are the sensitivities and specificities of the tests used to diagnose CDI [13]. In addition, the IDSA 2017 guidelines do not recommend repeat testing within seven days during the same diarrheal episode in symptomatic patients [10]. 

**Table 2:** The sensitivity and specificity of various testing modalities available clinically to diagnose CDI.

Methods/Assay	Sensitivity (% range)	Specificity (% range)
Cell Culture Cytotoxicity Neutralization Assay	33-86	97-100
Toxin A/B EIA	41-86	91-99
GDH EIA antigen	88-95	94-98
NAAT	62-100	89-100

In our case, the patient’s stool was sent for EIA toxin A and B and GDH antigen test, which is the standard of care. The EIA toxin A and/or B testing and GDH antigen testing were negative three times; however, clinical suspicion was still high. Historically, the gold standard for the diagnosis of CDI is the cell culture cytotoxicity neutralization assay (CCCN), however culturing of cells is labor-intensive, time-consuming, and not practical clinically [14]. Given our patient’s high clinical suspicion of CDI, the decision was made for a diagnostic colonoscopy which ultimately revealed pseudomembranous colitis. Pseudomembranes are only found in 13% of patients who have CDI and are typically suggestive of severe infection [8,9]. Although current guidelines do not recommend diagnostic colonoscopy in suspected CDI, a ​​systematic review by Kazanowski et al. concluded that there are certain criteria that indicate the need for endoscopic diagnosis in CDI [12]. They include negative laboratory tests with high clinical suspicion, failure to respond to medical treatment, atypical presentation of disease with evidence of obstruction and mild diarrhea, and when the diagnosis is needed before laboratory tests are available [12,15]. The patient in our presentation fell into the categories of high clinical suspicion with negative laboratory tests and a failure to respond to medical management. A confirmatory diagnosis was made after visualizing the pseudomembranes on the colonic mucosa and also ruled out other differential diagnoses such as microscopic colitis or gastroenteritis. 

While considering differentials for a patient presenting with a case of refractory diarrhea and multiple negative *C. difficile* EIA toxin A and B and GDH antigen, clinical suspicion for *C. difficile* colitis should remain high if there are underlying risk factors such as recent antibiotic use or recent hospitalizations as seen in our patient above [16,17]. If the patient has no absolute contraindication, then colonoscopic imaging should be done. What makes this case interesting is the fact that our patient got tested multiple times at two different facilities for *C. difficile* stool toxin and GDH antigen test but tested negative despite having colonoscopic evidence of diffuse pseudomembranous colitis. Once he was started on appropriate oral antibiotics for his CDI, his symptoms improved.

Other underlying factors such as laboratory error, and other underlying causes of pseudomembranous colitis such as inflammatory bowel disease, immune-mediated small-vessel vasculitis such as Behçet disease, and *Cytomegalovirus-*mediated colitis should also be investigated. A colonoscopy with biopsy may be needed to confirm the exact etiology causing the pseudomembranes as seen in our case above. Our patient had a positive biopsy revealing active CDI. The fact that our patient got better after he was started on oral vancomycin further confirms that *C. difficile *was* *the culprit of his pseudomembranes. The exact mechanism for why patients can have multiple negative two-step testing for CDI but still have endoscopic evidence of active *C. difficile-*mediated pseudomembranous colitis is still a conundrum and more research is needed for it to be elucidated. 

## Conclusions

Although pseudomembranes in the colon are non-specific, they are most commonly associated with *C. difficile* colitis especially in the appropriate clinical setting where there is a high index of suspicion. As seen in the case above, in rare instances patients can have multiple false negative tests for* C. difficile* due to unclear reasons. This can lead to inappropriate antibiotic use and treatment failure. Thus, it is necessary for clinicians to consider the use of direct visualization of the colon by colonoscopy in such cases, to facilitate accurate diagnosis and initiate prompt treatment in *C. difficile* at-risk populations. 
